# Storage of Sputum in Cetylpyridinium Chloride, OMNIgene.SPUTUM, and Ethanol Is Compatible with Molecular Tuberculosis Diagnostic Testing

**DOI:** 10.1128/JCM.00275-19

**Published:** 2019-06-25

**Authors:** C. N’Dira Sanoussi, Bouke C. de Jong, Dissou Affolabi, Conor J. Meehan, Mathieu Odoun, Leen Rigouts

**Affiliations:** a Laboratoire de Référence des Mycobactéries, Cotonou, Benin; b Unit of Mycobacteriology, Institute of Tropical Medicine, Antwerp, Belgium; c Department of Biomedical Sciences, University of Antwerp, Antwerp, Belgium; Carter BloodCare & Baylor University Medical Center

**Keywords:** AFB scanty, OMNIgene.SPUTUM, Xpert, cetylpyridinium chloride, ethanol, isolate, molecular tests, short/long-fragment PCR, sputum, storage

## Abstract

We compared cetylpyridinium chloride (CPC), ethanol (ETOH), and OMNIgene.SPUTUM (OMNI) for 28-day storage of sputum at ambient temperature before molecular tuberculosis diagnostics. Three sputum samples were collected from each of 133 smear-positive tuberculosis (TB) patients (399 sputum samples). Each patient’s sputum was stored with either CPC, ETOH, or OMNI for 28 days at ambient temperature, with subsequent *rpoB* amplification targeting a short fragment (81 bp, GeneXpert MTB/RIF [Xpert]) or a long fragment (1,764 bp, in-house nested PCR). For 36 patients, Xpert was also performed at baseline on all 108 fresh sputum samples. After the 28-day storage (D28), Xpert positivity did not significantly differ between storage methods. In contrast, higher positivity for *rpoB* nested PCR was obtained with OMNI (*n* = 125, 94%) than with ETOH (*n* = 114, 85.7%; *P* = 0.001). Smears with scanty acid-fast bacilli (AFB) had lower *rpoB* PCR positivity with ETOH storage (*n* = 10, 41.7%) than with CPC (*n* = 16, 66.7%; difference, 25%; 95% confidence interval [CI], 3.5 to 46.5; *P* = 0.031) or OMNI (*n* = 16, 69.6%; difference, 26.1%; 95% CI, 3.8 to 48.4; *P* = 0.031), with no difference between CPC and OMNI. Poststorage, the threshold cycle (*C_T_*) values significantly decreased compared to those prestorage with ETOH (difference, −1.1; 95% CI, −1.6 to −0.6; *P* = 0.0001) but not with CPC (*P* = 0.915) or OMNI (*P* = 0.33). For one patient’s ETOH- and CPC-stored specimens with a *C_T_* of <10, Xpert gave results of rifampin false resistant at D28, which was resolved by repeating Xpert on a 1/100 diluted specimen. In conclusion, 28-day storage of sputum in OMNI, CPC, or ETOH at ambient temperature does not impact short-fragment PCR (Xpert), including for low smear grades. However, for long-fragment PCR, ETOH yielded a lower PCR positivity for low smear grades, while the performance of OMNI and CPC was excellent for all smear grades. (The study has been registered at ClinicalTrials.gov under registration number NCT02744469.)

## Abstract (precis)

We compared cetylpyridinium chloride (CPC), ethanol (ETOH), and OMNIgene.SPUTUM (OMNI) for 28-day storage of sputum at ambient temperature before molecular tuberculosis diagnostics. Three sputum samples were collected from each of 133 smear-positive tuberculosis (TB) patients (399 sputum samples).

## INTRODUCTION

Molecular tuberculosis (TB) diagnostics are more sensitive than microscopy and faster than culture. In addition to the detection of TB, molecular analyses can provide the drug resistance profile of the affecting Mycobacterium tuberculosis complex (MTBC) strain(s) and strain typing for diversity studies. In low-income countries, molecular analyses may not be available in peripheral laboratories, and specimens need to be shipped to remote intermediate or central reference laboratories. Logistic constraints can delay the time between specimen collection and analysis, potentially negatively impacting the results.

Sample deterioration can be overcome by the use of sputum preservation reagents, such as ethanol (ETOH) (1) and cetylpyridinium chloride (CPC), already widely used, or a proprietary buffer such as OMNIgene.SPUTUM (OMNI; DNA Genotek, Ottawa, Canada) (2, 3). ETOH preservation allows for molecular analyses only, as it inactivates TB bacilli (1), while CPC and OMNI preservation can precede both molecular analyses and culture (2, 3). ETOH-preserved samples no longer constitute a biosafety risk, hence do not require a specific containment laboratory level, and can be shipped with minimal restrictions. ETOH is less expensive than CPC, which in turn is less expensive than OMNI.

No limit has been specified for specimen preservation in ETOH and CPC, while for OMNI, storage for a maximum of 8 days is specified by the manufacturer. The time between specimen collection and analysis can vary widely depending on shipping logistics and distance, the urgency of the molecular analysis to be performed, and whether specimens are assembled for batch processing. A recent systematic review highlighted the lack of evidence on the performance of commercial storage reagents to preserve sputum samples, especially paucibacillary samples, which is of great diagnostic importance (4, 5).

Molecular methods for the detection of rifampin (RIF), for example, vary by platform and target length, ranging from the 81-bp rifampin resistance-determining region (RRDR) target of the *rpoB* gene covered in GeneXpert MTB/RIF (Xpert; Cepheid, Sunnyvale, CA, USA) (6) to the 1,764-bp target of the *rpoB* gene that also covers resistance-conferring mutations positioned outside the RRDR (7).

We compared ETOH, CPC, and OMNI for 1-month (28-day) storage of smear-positive sputa at ambient temperature, with subsequent automated Xpert and conventional gel-based *rpoB* nested PCR, including paucibacillary specimens.

## MATERIALS AND METHODS

### Ethics considerations.

This evaluation was embedded in the BeniDiT study that was approved by the national ethics committee of Benin and the ethics committees of the Institute of Tropical Medicine (ITM) of Antwerp and the University of Antwerp in Belgium. The study was registered at ClinicalTrials.gov under registration number NCT02744469. Before inclusion, all the study participants provided written informed consent. Participants’ specimens were pseudonymized before laboratory analyses.

### Study design, participants, and specimens.

**FIG 1:**
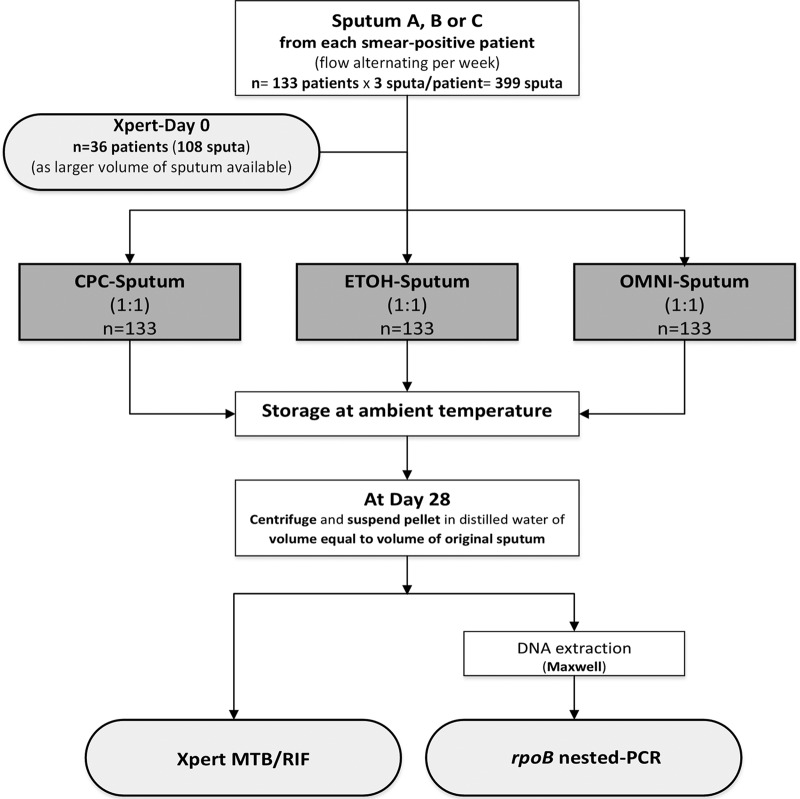
Flow diagram for specimens and methods.

The main study design and specimen workflow are summarized in Fig. 1. In total, 399 sputum samples were collected from 133 consecutive new smear-positive TB patients who were prospectively recruited among patients registered for TB screening in the National University Hospital for TB and Lung Diseases in Cotonou, Benin (Centre National Universitaire Hospitalier de Pneumo-Phtisiologie [CNHU-PPC]) during a 3-month period. The sputa were collected before patients started anti-TB treatment. Laboratory analyses were performed in the supranational reference laboratory for mycobacteria in Cotonou, Benin (Laboratoire de Référence des Mycobactéries [LRM]), located in the CNHU-PPC.

In total, three pretreatment sputum samples (spot day 1, morning, and spot day 2) were collected from each participating patient. TB confirmation, per national guidelines (8), was based on smear microscopy on unprocessed sputa using the WHO/IUATLD acid-fast bacillus (AFB) grading scale for fluorescence microscopy (9). Each sputum sample was stored with either an equal volume of ETOH (final concentration 50%), CPC 1% (final 0.5%), or OMNI (final 50%) (1, 3). The 1% CPC solution was prepared by dissolving the necessary amount of CPC in a 2% sodium chloride solution. The sequence of the sputum samples (spot day 1, morning, and spot day 2) assigned to a specific storage reagent alternated weekly. The sputum mixtures were vortexed and stored at ambient temperature for 28 days, after which they were centrifuged at 3,800 × *g* (3) for 20 min at 28°C, the supernatant was discarded, and the pellet was resuspended in sterile distilled water (of equal volume to the initial unprocessed sputum). At least 1 ml of the suspension was used for Xpert and 200 μl was extracted for *rpoB* nested PCR. For 36 patients (108 sputum samples) who produced large-volume samples (at least 2.5 ml per sputum container), the Xpert analysis was also performed at baseline before storage of the sputum in the storage reagent. The ambient temperature of the storage room was recorded every day; no temperature control measures such as air conditioning were in place.

Prior to the main study, at the Institute of Tropical Medicine in Antwerp (ITM), the reference M. tuberculosis isolate H37Rv (dilution 10^−3^ McFarland 1) was also stored in ETOH, CPC, and OMNI, in triplicates, for 37 days at ambient temperature and 37°C and tested with Xpert to check whether the detectable bacillary load would be affected. As the ITM laboratory is air conditioned (20°C to 25°C) and Belgium has a moderate climate, the standard set temperature of the incubator (37°C) was used to mimic higher temperature conditions encountered in many countries of high endemicity. To mimic delayed intercontinental shipments, such as those potentially facing customs clearance issues, the storage time was extended to 37 days.

### Molecular analyses.

Xpert MTB/RIF is an automated system with DNA extraction and amplification integrated for the real-time quantitative PCR (qPCR) of five probes (A, B, C, D, and E) covering the RRDR of the *rpoB* gene. Resistance to RIF is detected through absence or delayed reaction with one or more probes (6). The assay was used as per the manufacturer’s instructions (6). For the fresh sputum (when baseline Xpert was performed before storage) or for the suspension after storage, one volume of the specimen (suspension-sputum) was mixed with 2 volumes of the Xpert “sample reagent” as recommended by the manufacturer for sputum, to ensure the comparability of fresh sputum and respective suspension after storage. Poststorage threshold cycle (*C_T_*) values were compared across storage methods and to *C_T_* values from fresh sputum when available. To ensure valid comparisons, specimens and Xpert sample reagent volumes were measured with graduated pipets.

### In-house *rpoB* nested PCR.

Sediments from CPC- and OMNI-stored specimens were inactivated at 95°C for 10 min (10). After a digestion of 200 μl of the suspension with proteinase K, DNA was extracted using the semiautomated Maxwell 16 tissue DNA purification kit (AS1030) with a Maxwell machine (model 4.9; Promega) and eluted in 300 μl Maxwell elution buffer as previously described (11, 12). *rpoB* nested PCR was performed as previously described (7). Positive and negative extraction as well as amplification controls were included. The positive extraction control was a sediment from a sputum known as MTBC PCR positive. The positive amplification controls consisted of DNA extracts from H37Rv suspensions (10^−2^, 10^−3^, and 10^−4^ of McFarland 1) and DNA extracts (10^−2^ and 10^−4^ μg/μl) from MTBC PCR-positive sputum. The negative extraction control consisted of molecular-grade water processed as other sputum specimens in each DNA extraction run. The negative amplification control consisted of molecular-grade water (for both 1st PCR run to nested run and nested run separately) processed as a specimen DNA extract along with DNA extracts from sputum. All specimens (ETOH, CPC, and OMNI) from a patient were processed in the same extraction run or PCR run. For the conventional PCR, specimens were coded so that the person reading the gel was blinded to the patient’s identification (ID) or storage method assigned. To check for possible PCR inhibition, the *rpoB* nested PCR was repeated for specimens with a negative result using a one-tenth dilution of the original DNA extract.

### Statistical analyses.

Statistical data analysis was performed using Stata 12.0 (StataCorp, USA). We used McNemar’s chi-square test to compare paired categorical data across ETOH, CPC, and OMNI groups and paired *t* test to compare *C_T_* values. The difference (Diff) in proportion or mean difference was calculated with a 95% confidence interval (CI) and *P* value, which was considered significant at <0.05.

## RESULTS

The sputa stored in the respective reagents had similar AFB positivity grades (see Table S1 in the supplemental material). In the room where sputum mixtures with storage reagent were stored, the temperature ranged from 26°C to 31.9°C, with an average of 28.5°C and a median of 28.4°C. All included negative controls remained negative by nested PCR.

After the 28 days of storage (D28), the presence of MTBC was confirmed by Xpert in 132 (99.2%) patients. Xpert yielded a positive result for most sputa (98.5%, 99.2%, and 99.2% for ETOH, CPC, and OMNI, respectively), and the Xpert positivity across storage methods did not significantly differ (Table 1). After stratification by AFB smear grading, all but three AFB-scanty sputum samples (2 stored with ETOH, 1 stored with CPC) and a 1+ AFB-positive sputum (stored with OMNI) were Xpert positive (see Table S2). However, also for scanty specimens, positivity was not significantly different across storage methods for Xpert (Table S2).

**TABLE 1:** Positivity of PCR by storage method

Storage	Xpert MTB/RIF at D28				*rpoB* PCR at D28			
	No. (%) positive	Total no.	Diff (95% CI)^*a*^	*P* value^*b*^	No. (%) positive	Total no.	Diff (95% CI)^*a*^	*P* value^*b*^
ETOH-28	131^*c*^ (98.5)	133			114^*e*^ (85.7)	133		
CPC-28	132 (99.2)	133	0.8 (−1.5 to 3) A	1	121^*e*^ (91)	133	5.3 (−1.1 to 11.7) A	0.119
OMNI-28	132^*d*^ (99.2)	133	0.8 (−1.5 to 3) B	1	125^*e*^ (94)	133	8.3 (2.8 to 13.7) B	0.001
OMNI-28 vs CPC-28			0 (−0.8 to 0.8) C	1			3 (−1.9 to 7.9) C	0.289
Total	395 (99)	399			360 (90.2)	399		

a Uppercase letters indicate comparisons for statistical testing. A, CPC-28 versus ETOH-28; B, OMNI-28 versus ETOH-28; C, OMNI-28 versus CPC-28.

b *P* values were calculated using McNemar’s exact test.

c Includes 2 errors that became positive (high) after testing 1/10 dilution of the sediment.

d Includes 1 invalid that became positive (very low) after testing 1/10 dilution of the sediment.

e For all PCR-negative specimens, including a set of specimens with discrepant PCR results from the same patient, the *rpoB* PCR was repeated on a 1/10 dilution of the DNA extract. All remained negative, but one stored in OMNI became positive.

In contrast, higher positivity for *rpoB* nested PCR was obtained with OMNI storage (94%), which differed significantly between OMNI and ETOH (*P* = 0.001) (Table 1). Most negative nested PCRs occurred among AFB-scanty sputa (14/24 for ETOH, 9/25 for CPC, and 7/24 for OMNI) (Table 2). Lower *rpoB* PCR positivity among AFB-scanty sputa was found with ETOH storage than with either CPC (Diff, 25%; 95% confidence interval [CI], 3.5 to 46.5; *P* = 0.031) or OMNI (Diff, 26.1%; 95% CI, 3.8 to 48.4; *P* = 0.031), whereas CPC and OMNI storage yields were similar (*P* = 1) (Table 2; see also Table S3). For 1+ AFB-positive sputa, the differences between ETOH, CPC, and OMNI were not significant (Table S3).

**TABLE 2:** Positivity of *rpoB* PCR by sputum AFB grade

Sputum AFB grade^*a*^	*rpoB* PCR results for specimens stored in:					
	ETOH		CPC		OMNI	
	No. (%) positive	Total no.	No. (%) positive	Total no.	No. (%) positive	Total no.
3+ positive	13 (100)	13	16 (100)	16	15 (100)	15
2+ positive	63 (96.9)	65	63 (98.4)	64	68 (100)	68
1+ positive	28 (90.3)	31	26 (92.9)	28	25 (96.2)	26
Scanty^*b*^	10 (41.7)	24	16 (64)	25	17 (70.8)	24
Total	114	133	121	133	125	133

a UIATLD/WHO scale for fluorescence microscopy (9) using original (fresh unprocessed) sputum.

b Among smear-scanty specimens, Xpert positivity was significantly different for CPC versus ETOH (McNemar’s exact test, *P* = 0.31) and OMNI versus ETOH (*P* = 0.31) but similar for OMNI versus CPC (*P* = 1) (see Table S3 in the supplemental material).

In total, sputa from two patients tested RIF resistant in Xpert, with probes A or E absent. Hence, we compared *C_T_* values of probe B and D to assess *C_T_* value variability across storage methods. The mean *C_T_* values for probe D after 28-day storage were 17 for ETOH, 17.9 for CPC, and 18.1 for OMNI. The probe D *C_T_* values after ETOH storage were significantly lower (more MTBC DNA amplified) than those observed either after CPC storage (CPC versus ETOH, *P* < 0.00001) or after OMNI storage (OMNI versus ETOH, *P* < 0.00001) but did not significantly differ between OMNI and CPC storage (*P* = 0.51) (Table 3). Likewise, for probe B, *C_T_* values were significantly lower for ETOH than CPC or OMNI storage, but no difference was observed for OMNI versus CPC (data not shown).

**TABLE 3:** Change in Xpert *C_T_* values for probe D after 28-day storage across storage methods

Storage method^*a*^	Total no.	Xpert D28 probe D *C_T_* values		*P* value^*b*^	
		Mean (95% CI)	Diff (95% CI)	Diff = 0	Ha: Diff > 0
ETOH-28	131	17.0 (16.3–17)			
CPC-28	131	17.9 (17.2–18)	0.9 (0.5 to 1.3)	<0.00001	<0.00001
ETOH-28	130	17.0 (16.4–17.7)			
OMNI-28	130	18.1 (17.4–18.8)	1.1 (0.6 to 1.6)	<0.00001	<0.00001
CPC-28	131	18 (17.3–18.7)			
OMNI-28	131	18.2 (17.5–18.8)	0.2 (−0.3 to 0.7)	0.51	0.253

a ETOH-28, storage in ETOH for 28 days; CPC-28, storage in CPC for 28 days; OMNI-28, storage in OMNI for 28 days.

b *P* values were calculated using paired *t* tests; only paired sputa (i.e., sputa from the same patient) with positive Xpert (i.e., *C_T_* ≠ 0) for the 2 compared storage methods were included. Ha, alternative hypothesis for the statistical analysis.

As a sensitivity analysis, for 36 patients (108 sputum samples) for whom Xpert was also performed on fresh sputum, we compared *C_T_* values for three fresh unprocessed (D0) and three poststorage (D28) sputum samples across storage methods. No significant difference in D0 *C_T_* values was observed between the sputa assigned to either ETOH, CPC, or OMNI storage (mean D0 *C_T_*s for probe D, 18.6 for ETOH versus 18.4 for CPC versus 18.1 for OMNI; nonsignificant for all comparisons), showing that groups of sputa assigned to each of the three storage methods were comparable in terms of fresh bacillary load before mixing with storage reagents. However, overall D28 *C_T_* values compared across storage methods were significantly lower for ETOH than for CPC or OMNI yet not different for OMNI versus CPC (mean D28 *C_T_*s for probe D, 17.5 for ETOH versus 18.3 for CPC versus 18.8 for OMNI, with *P*_(CPC-ETOH)_ = 0.015; *P*_(OMNI-ETOH)_ = 0.005; *P*_(OMNI-CPC)_ = 0.31). This confirmed the findings on all sputa that ETOH preservation, unlike CPC or OMNI, lowers Xpert *C_T_* values. Despite the fact that the quantification of the bacterial load by sputum AFB microscopy is less precise than by Xpert *C_T_* value, ETOH storage also yielded more Xpert “high” bacillary loads among AFB weakly positive (1+ positive and scanty) sputa (25.5% versus 11.3% for CPC and 6% for OMNI) (Table S2).

Likewise, a direct comparison of *C_T_* values from baseline and stored sputum from the same patient (*C_T_*_(stored [D28])_ − *C_T_*_(fresh [D0])_) significantly decreased (more detectable DNA) after storage in ETOH (−1.1; 95% CI, −1.6 to −0.6; *P* = 0.0001 for probe D), whereas it did not differ after storage in CPC (*P* = 0.915, probe D) or OMNI (*P* = 0.33, probe D) (Table 4 for probe D; see Table S4 for probe B).

**TABLE 4:** Change in *C_T_* values for Xpert probe D from storage at D0 to processing at D28

Storage^*a*^	Total no. of specimens	Probe D *C_T_* values		*P* value^*b*^	
		Mean (95% CI)	Diff (95%CI)	Ha: Diff < 0	Diff = 0
ETOH					
D0	36	18.6 (17.3–19.9)			
D28	36	17.5 (16.1–19)	−1.1 (−1.6 to −0.6)	0.0001	0.0002
CPC					
D0	36	18.4 (17–19.8)			
D28	36	18.3 (16.8–19.9)	−0.03 (−0.7 to 0.5)	0.457	0.915
OMNI					
D0	35^*c*^	18.1 (16.8–19.4)			
D28	35^*c*^	18.3 (17–19.7)	0.2 (−0.3 to 0.7)	0.835	0.330

a D0, day 0 (baseline before storage with a storage reagent); D28, after 28-day storage with a storage reagent.

b *P* values were calculated using paired *t* tests. Ha, alternative hypothesis for the statistical analysis.

c One sputum excluded from that *C_T_* difference analysis on OMNI, as the AFB-positive sputum (of the 3 AFB-scanty sputa of a patient) randomly assigned to OMNI had a negative D0 Xpert.

For one patient, all three baseline sputum samples (D0) were RIF sensitive by Xpert. At D28, the ETOH-stored specimen turned RIF resistant, while CPC- and OMNI-stored sputa remained RIF sensitive. After repeat testing of the three D28 specimens by Xpert, RIF resistance was confirmed for the ETOH specimen (probe A and E missing, *C_T_* of 8.7 for probe D) and the CPC specimen (only probe A missing, *C_T_* of 8.7 for probe D), while the OMNI specimen remained RIF sensitive (*C_T_* of 11.5 for probe D). As the Xpert *C_T_* values were low, we repeated Xpert on a 1/100 dilution of the D28 suspensions, and all were RIF sensitive. RIF sensitivity was further confirmed by testing the undiluted suspensions with Xpert Ultra (Cepheid, Sunnyvale, CA, USA) and DNA sequencing of the *rpoB* nested PCR amplicons (wild-type *rpoB*).

Regarding the H37Rv bacterial suspensions, the Xpert positivity was the same (“medium”) for CPC and OMNI at either ambient temperature or 37°C, yet for ETOH, the results were temperature dependent, remaining “medium” at ambient temperature but decreasing (“low”) at 37°C in two replicates.

## DISCUSSION

ETOH, CPC, and OMNI storage for 28 days did not yield significant differences in the proportions that tested Xpert positive, whereas successful amplification of a large *rpoB* target was significantly more likely after OMNI storage (94%) than after ETOH storage (85.7%), without significant difference with CPC (91%). Of concern, one sample tested false RIF resistant after 28-day storage in ETOH and CPC, likely related to the lowered *C_T_* values after storage, below the range for optimal detection of rifampin resistance. Thus, for all sputa—whether fresh or stored—in the context of *C_T_* values of ≤10, a RIF resistant result should be confirmed by dilution (1/100) of the remaining sample or a new sample if there are none left over. Studies similar to this could be conducted on Xpert Ultra as this becomes the standard Xpert MTB/RIF testing (13).

Other authors comparing shorter storage durations (2 to 21 days) also found comparable Xpert positivity after storage in CPC (98.9% versus 99.2% in our study) or OMNI (97.9% [14] and 95% [15] versus 99.2% in our study).

The yields of a long *rpoB* fragment from AFB-scanty sputa were significantly higher for OMNI and CPC storage than for ETOH storage. On the contrary, Xpert *C_T_* values after the 28-day storage were significantly lower in ETOH-stored sputa than those stored in CPC or OMNI, despite comparable *C_T_* values in fresh sputa at baseline. Similarly, Asandem et al. found that OMNI yielded less Xpert “high” bacillary load (after 7 days of storage) versus that in freshly decontaminated sediments (decrease of 6%) (14). The differences observed for ETOH storage, with lower yields for long-fragment PCR yet higher bacterial loads detected in Xpert than with CPC and OMNI, are unlikely to be explained by a lower compatibility of ETOH with Maxwell DNA extraction, as ETOH enhances/facilitates DNA extraction (as also shown by Rabodoarivelo et al. [16]) by destroying the protein structure of the bacterial cell surface (17). This was confirmed by the lowered *C_T_* at D28 for EtOH samples in our study. Furthermore, the storage reagent mixed with the sputum was discarded after the poststorage centrifugation, before the pellet was resuspended in sterile distilled water, thus limiting the interaction between storage and extraction reagents. Also, such interaction between ETOH and proteinase K in lysing buffer (pH 7.5) used prior to our modified Maxwell extraction is unlikely (11, 18). In addition, ETOH is a component of the Maxwell extraction cartridge (security datasheet [12]). The more plausible explanation is the fragmentation/degradation of the DNA, as ETOH creates a disordered state in the bacterial DNA by cross-linking the bases so that the DNA strands can no longer properly separate, interfering with replication and transcription (17). Therefore, the initial denaturation step of the PCR process might cause fragmentation of the bacterial DNA, which no longer properly separates, as result of the ETOH effect. This could affect the recovery/amplification of the bacterial DNA, which is more perceivable in a long-fragment target (1,764 bp in *rpoB* PCR versus 81 bp in Xpert), especially in paucibacillary samples. Likewise, NaOH-induced changes in the bacterial DNA sequences of paucibacillary samples after prolonged storage in Xpert sample reagent risk causing false resistant results (19). Furthermore, the impact of ETOH storage might be temperature dependent, as evidenced by data of the H37Rv isolate with decreased positivity at 37°C. This needs to be further investigated, as we only tested one strain, albeit in triplicates, and our sputum samples were not exposed to temperatures above 32°C.

Storage of sputum in 50% (used in this study) or 70% ETOH final concentration (used in some laboratories) renders the TB bacilli nonviable after 1 h of storage (1; unpublished data from our laboratory [ITM]). PCR results for a 123-bp fragment were similar for both ETOH concentrations after 1 day of storage (1). The long-fragment *rpoB* PCR after 1-h storage of 4 triplicates of isolate-spiked sputa yielded 12 positive results in 50% ETOH versus 11 in 70% ETOH and, after 14-days storage, 10 versus 11 positives, respectively (unpublished data from ITM). In other unpublished data from ITM, analyses of 943 sputa after long-term (20 to 218 days) storage in ∼70% ETOH at noncontrolled “ambient” temperature in Bangladesh showed a similar positivity in *rpoB* nested PCR for 3+, 2+, and 1+ AFB-grade sputa (96%, 95%, and 90% versus 100%, 96.9%, and 90.3%, respectively, in the current study). In contrast, positivity among AFB-scanty sputa was lower in the present study (41.7%) than in the unpublished data (77%). Van Deun et al. found that sputa stored in 70% ETOH for a period ranging from 2 to 7 years yielded an overall positivity of 94% in the long-fragment *rpoB* nested PCR versus 85.7% for 50% ETOH in this study (20).

A strength of this study is the paired design, with comparable bacterial loads by AFB smear-positivity grading and Xpert *C_T_* values between all groups. As a limitation, ambient temperatures vary, potentially affecting the generalizability of our findings to hotter climates, and we did not test long-term storage without additives, which may be challenging because of potential overgrowth by other organisms. Smear-negative sputa from TB patients were not included in this study, with likely further decreased yield of *rpoB* amplification in all storage solutions. Moreover, we did not test the compatibility of different storage solutions with other DNA extraction methods other than the Maxwell method. The use of basic extraction methods consisting of boiling sputum (such as the Chelex method) resulted in good yield after storage of sputum in ETOH (16). We centrifuged and discarded the storage reagent from sputa before testing (as recommended for OMNI [3]) to avoid eventual reagent interaction, which is unlikely for ETOH and unknown for CPC and OMNI. This may not be feasible in Xpert centers without centrifuges. We centrifuged stored sputa at 3,800 × *g*, the upper range (3,000 to 3,800 × *g*) recommended for OMNI by its manufacturer (3) and compatible with other TB analyses (≥3,000 × *g* recommended [9]). At 3,000 × *g* but not 3,800 × *g*, we noted that the OMNI pellet tended to slip and be discarded with supernatant after centrifugation.

**FIG 2:**
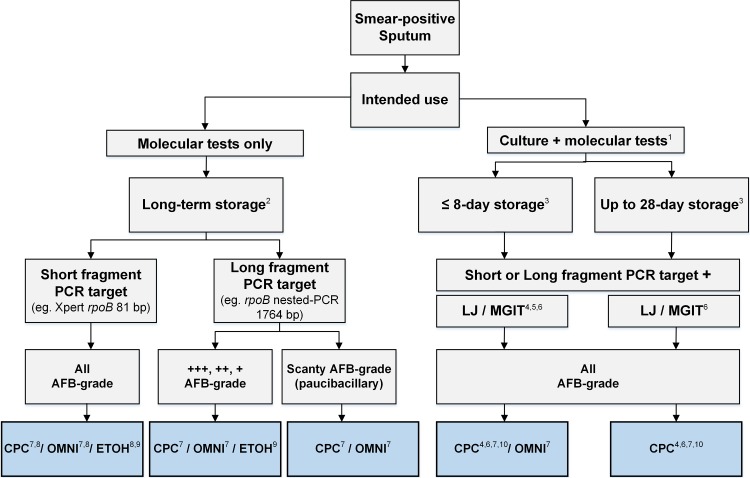
Algorithm for the choice of the suitable reagent for the storage of sputum for TB diagnostics. 1, Molecular tests on the stored sputum (direct molecular tests); 2, from up to 28 days (present study) to 2 to 7 years (20); 3, previously showed by Affolabi et al. (21); 4, extra washing required before inoculation on culture medium (2, 21, 25); 5, reduced/delayed growth after OMNI storage (2, 22, 23); 6, CPC is not compatible with direct MGIT inoculation (24), with reduced/delayed positivity (25), but washing off the CPC prior to inoculation in MGITs (2, 21, 25) increases the culture positivity rate (25); 7, cost OMNI > CPC > ETOH, shipping restriction for specimens stored with CPC and OMNI (IATA category B); 8, when Xpert *C_T_* values are <10, a RIF-resistant result should be confirmed on a dilution (1/100) of the remaining sample or a new sample if no leftover remains; 9, not optimal with higher temperature (≥37°C tested in this study), ETOH has shipping restrictions: dangerous good if total volume is >100 ml; 10, short decontamination required before inoculation on culture medium (21); LJ, Löwenstein-Jensen medium; MGIT, mycobacterial growth indicator tube (manual or automated); LJ/MGIT, for LJ or MGIT; CPC/OMNI/ETOH, use either CPC, OMNI, or ETOH; CPC/OMNI, use either CPC or OMNI.

As summarized in Fig. 2, in conclusion, the overall performance of ETOH, CPC, and OMNI for 28-day storage of sputum at ambient temperature is excellent, especially for subsequent Xpert analysis and long-fragment PCR of non-scanty AFB-positive samples. It is advisable to use ETOH for subsequent short-fragment PCR (Xpert) when culture is not needed and to use either CPC or OMNI when culture is needed. For paucibacillary sputa, OMNI or CPC will have better yields for subsequent long-fragment PCR. When culture is needed, the choice between OMNI and CPC, which are equally performant for either subsequent short-fragment PCR (Xpert) or long-fragment (conventional) PCR, should be based on their cost (OMNI is ∼57× more expensive than CPC [∼$1.15 versus ∼$0.02/ml of sputum] [5]) and their performance for culture after 28-day storage (CPC followed by a short decontamination is better than OMNI for culture on Lowenstein-Jensen medium [21]). The choice should be also based on the culture medium to be used. There is a reduced/delayed growth in mycobacterial growth indicator tubes (MGITs) after OMNI (2, 22, 23). Likewise, direct inoculation of CPC-preserved specimens is not compatible with MGITs (24), with a reduced/delayed positivity (25); however, washing off the CPC prior to inoculation in MGITs (2, 21, 25) increases the culture positivity rate (25), a procedure not yet common in routine practice. One should also take into account whether poststorage fluorescence microscopy-based tests will be realized (CPC is not compatible with fluorescence microscopy [2]), the constraints regarding import or transport of dangerous products (CPC), the availability of a centrifuge adjustable to ambient temperature (CPC crystalizes at cold temperatures [9]), and the availability of a centrifuge reaching 3,800 × *g* (needed for OMNI). CPC and OMNI are likely more stable than ETOH for the storage of specimens/cultures at high ambient temperature (≤37°C) and possibly beyond.

## Supplementary Material

Supplemental file 1
