# Supplementary material for: Storage of Sputum in Cetylpyridinium Chloride, OMNIgene.SPUTUM, and Ethanol Is Compatible with Molecular Tuberculosis Diagnostic Testing
Source: J Clin Microbiol. 2019 Jun 25;57(7):e00275-19. doi: 10.1128/JCM.00275-19 (PMC6595455; doi:10.1128/JCM.00275-19)
Supplement: Supplemental file 1 [file JCM.00275-19-s0001.pdf]

Table S1. AFB-grading of sputa by storage method

**Sputa# assigned to**	**AFB-grade (auramine)‡#**											
	**+++ Pos**	**Diff (95%CI)**	**P**	**++ Pos**	**Diff (95%CI)**	**p**	**+ Pos**	**Diff (95%CI)**	**p**	**Scanty**	**Diff (95%CI)**	**p**
**ETOH**	13 (9.8)	-		65 (48.9)	-		31 (23.3)	-		24 (18.0)	-	
**CPC**	16 (12.0)	2.3(-1 to 5.5)[A]	0.25	64 (48.1)	-0.7(-5.9 to 4.4)[A]	1	28 (21.1)	-2.3(-6.9 to 2.4)[A]	0.453	25 (18.8)	0.8(-1.5 to 3)[A]	1
**OMNI**	15 (11.3)	1.5(-2.2 to 5.2)[B]	0.625	68 (51.1)	2.3(-2.9 to 7.4)[B]	0.51	26 (19.5)	-3.8(-9.8 to 2.3)[B]	0.267	24 (18.0)	0(-2.8 to 2.8)[B]	1
**OMNI vs CPC**		-0.7(-5.4 to 3.9)[C]	1		3(-3.2 to 9.2)[C]	0.424		-1.5(-6.9 to 3.9)[C]	0.754		-0.8(-3 to 1.5)[C]	1
Total	44			197			85			73		

‡UIATLD/WHO scale for fluorescent microscopy (1)

Original (fresh unprocessed) sputum; Diff: difference

p-values were calculated using Exact Mc Nemar’s Chi2 test, comparing (A) sputa assigned to (to be stored with) CPC versus those assigned to ETOH, (B) those assigned to OMNI versus ETOH and (C) those assigned to OMNI versus CPC.

Table S2. Positivity and bacilli load of Xpert by sputum AFB-grade

Sputum AFB-grade‡#	Xpert																	
	ETOH						CPC						OMNI					
	H n(%)	M n(%)	L n(%)	VL n(%)	All pos n(%)	Total n	H n(%)	M n(%)	L n(%)	VL n(%)	All pos n(%)	Total n	H n(%)	M n(%)	L n(%)	VL n(%)	All pos n(%)	Total n
**+++pos**	13 (100)	0	0	0	**13** (100)	13	15 (93.8)	1	0	0	**16** (100)	16	15 (100)	0	0	0	**15** (100)	15
**++pos**	51 (**78.5**)	14 (21.5)	0	0	**65** (100)	65	38 (59.4)	26 (40.6)	0	0	**64** (100)	64	40 (58.8)	27 (39.7)	0	1	**68** (100)	68
**+pos**	11 (**35.5**)	18 (58.1)	2 (6.5)	0	**31** (100)	31	5 (17.9)	21 (75)	2 (7.1)	0	**28** (100)	28	3 (11.5)	19 (73.1)	3 (**11.5**)	0	**25** (96.2)	26
**Scanty***	3 (**12.5**)	9 (37.5)	10 (41.7)	0	**22** (91.7)	24	1 (**4**)	9 (36)	14 (**56**)	0	**24** (96)	25	0 (**0**)	12 (**50**)	11 (45.8)	1	**24** (100)	24
**Total**	78	41	12	0	131	**133**	59	57	16	0	132	**133**	58	58	14	3	132	**133**
**pos+ and scanty**	14 (**25.5**)	25 (45.5)	12 (21.8)	0	53 (96.4)	55	6 (11.3)	30 (56.6)	16 (30.2)	0	52 (98.1)	63	3 (6)	31 (62)	14 (28)	1 (2)	49 (98)	50

H: high (Ct<16); M: medium (Ct 16-22); L: low (Ct 22 - 28); VL: very low (Ct >28); pos: positive

‡UIATLD/WHO scale for fluorescent microscopy (1)

Original (fresh unprocessed) sputum

*Among smear-scanty specimens, Xpert positivity was similar (Mc Nemar exact, p=1 for CPC vs ETOH, p=1 for OMNI vs ETOH, p=1 for OMNI vs CPC

Table S3. Pairwise comparison of storage methods for *rpoB* PCR positivity among lower grade AFB positive sputa (scanty and AFB-positive +)

***rpoB* PCR**

Scanty AFB

CPC-28 vs ETOH-28

	**ETOH +**	**ETOH -**	**Total**	**Diff(95%CI) p***
**CPC +**	10	**6**	16	**25 (3.5 to 46.5)**
**CPC -**	0	8	8	**0.031**
Total	10	14	24#	

OMNI-28 vs ETOH-28

	**ETOH +**	**ETOH -**	**Total**	**Diff(95%CI) p***
**OMNI +**	10	**6**	16	**26.1 (3.8 to 48.4)**
**OMNI -**	0	7	7	**0.031**
Total	10	13	23#	

OMNI vs CPC

	**CPC +**	**CPC -**	**Total**	**Diff(95%CI) p***
**OMNI +**	14	**3**	17	4.2 (-18.2 to 26.5)
**OMNI -**	**2**	5	7	1
Total	16	8	24#	

AFB positive +

CPC-28 vs ETOH-28

	**ETOH +**	**ETOH -**	**Total**	**Diff(95%CI) p***
**CPC +**	21	**3**	24	3.8 (-16.8 to 24.5)
**CPC -**	2	0	2	1
Total	23	3	26#	

OMNI-28 vs ETOH-28

	**ETOH +**	**ETOH -**	**Total**	**Diff(95%CI) p***
**OMNI +**	21	**2**	23	8.7 (7.2 to 24.6)
**OMNI -**	0	0	0	0.5
Total	21	2	23#	

OMNI vs CPC

	**CPC +**	**CPC -**	**Total**	**Diff(95%CI) p***
**OMNI +**	20	**2**	22	9.1 (-7.5 to 25.6)
**OMNI -**	**0**	0	0	1
Total	20	2	22#	

Specimens (from a same patient) and with the same AFB smear grading (scanty or positive+ accordingly) for the (two) storage methods compared were included in the analysis.

*****p-values were calculated with the Mc Nemar Exact test. **Diff**: difference

Table S4. Change in Ct-value for Xpert probe B from storage at D0 to processing at D28

		Total (per storage method)	Mean Ct_{probeB} (95%CI)	Diff Ct_{D28-D0} (95%CI)	p[#]
**ETOH**	D0	36	19.8 (18.6 to 21)	-	
	D28	36	**18.2 (16.9 to 19.6)**	**-1.5 (-2.1 to -0.9)**	**<0.00001**
**CPC**	D0	36	19.4 (18.1 to 20.7)	-	
	D28	36	19 (17.4 to 20.5)	-0.4 (-1.1 to 0.2)	0.247
**OMNI**	D0	35*	19.2 (17.9 to 20.5)	-	
	D28	35*	19.1 (17.8 to 20.4)	-0.1 (-0.7 to 0.4)	0.646

#p-values were calculated using paired t-test.

*One sputum excluded from that Ct difference analysis on OMNI as the AFB-positive sputum (of the 3 AFB-scanty sputa of a patient) randomly assigned to OMNI had a negative D0 Xpert.

REFERENCES FOR SUPPLEMETAL MATERIALS

1. Rieder H L VA, Kam K M , Kim S J, Chonde T M , Trébucq A , Urbanczik R. Second edition 2007. Priorities for Tuberculosis Bacteriology Services in Low-Income Countries, Second edition ed. International Union Against Tuberculosis and Lung Disease (The Union)
